# Obesity and Gray Matter Volume Assessed by Neuroimaging: A Systematic Review

**DOI:** 10.3390/brainsci11080999

**Published:** 2021-07-28

**Authors:** Marina Fernández-Andújar, Ester Morales-García, Natalia García-Casares

**Affiliations:** 1 Facultad de Psicología, Universitat Abat Oliba CEU, CEU Universities, 08022 Barcelona, Spain; mfernandezan@uao.es; 2 Servicio de Neurología, Hospital Universitario 12 de Octubre, 28041 Madrid, Spain; estermoralesgarcia@gmail.com; 3 Department of Medicine, Faculty of Medicine, University of Malaga, 29010 Malaga, Spain; 4 Centro de Investigaciones Médico-Sanitarias (C.I.M.E.S), University of Malaga, 29010 Malaga, Spain; 5 Área de Enfermedades cardiovasculares, obesidad y diabetes, Instituto de Investigación Biomédica de Málaga (IBIMA), 29010 Malaga, Spain

**Keywords:** obesity, adiposity, voxel-based morphometry, MRI, CT, atrophy, GMV

## Abstract

Obesity has become a major public and individual health problem due to its high worldwide prevalence and its relation with comorbid conditions. According to previous studies, obesity is related to an increased risk of cognitive impairment and dementia. This systematic review aims to further examine the present state of the art about the association between obesity and gray matter volume (GMV) as assessed by magnetic resonance imaging (MRI). A search was conducted in Pubmed, SCOPUS and Cochrane of those studies released before 1 February 2021 including MRIs to assess the GMVs in obese participants. From this search, 1420 results were obtained, and 34 publications were finally included. Obesity was mainly measured by the body mass index, although other common types of evaluations were used (e.g., waist circumference, waist-to-hip ratio and plasma leptin levels). The selected neuroimaging analysis methods were voxel-based morphometry (VBM) and cortical thickness (CT), finding 21 and 13 publications, respectively. There were 30 cross-sectional and 2 prospective longitudinal studies, and 2 articles had both cross-sectional and longitudinal designs. Most studies showed a negative association between obesity and GMV. This would have important public health implications, as obesity prevention could avoid a potential risk of GMV reductions, cognitive impairment and dementia.

## 1. Introduction

Obesity has become a major public health concern worldwide. In 2016, 39% of adults aged 18 years and older were overweight, and of these, over 13% were obese [1]. These epidemiological data show that obesity rates have more than doubled within the last forty years [1]. However, it is expected that one in four adults will have severe obesity in the United States by 2030 [2,3]. It is also a serious individual health problem; it is associated with the increased incidence and prevalence of numerous comorbid conditions, including hypertension, hyperlipidemia, type 2 diabetes, cardiovascular disease, certain types of cancer and musculoskeletal disorders as it affects most organ systems, including the central nervous system [4,5]. Previous studies have demonstrated that obesity is associated with lower cognitive performance (for instance, in relation with executive function, memory, processing speed or attention) [6,7,8,9] and higher risk of mild cognitive impairment and dementia, such as Alzheimer’s disease [10,11,12]. Alzheimer’s disease is a neurodegenerative disorder characterized by a progressive decline in cognitive function and a specific pattern of gray matter volume (GMV) reductions which can be assessed by neuroimaging. These structural alterations start years before symptoms appear and atrophy measures change with the progression of the disease, following a posterior-to-anterior gradient. In an early stage, the entorhinal cortex and hippocampus, structures located in the medial temporal lobe, are affected, producing early memory deficits. Subsequently, atrophy in the temporal, parietal and frontal neocortices is associated with memory, executive functions, language, visuospatial and behavioral impairments [13,14]. In patients with established Alzheimer’s disease, the atrophy rates are greater in all cortical regions with relative sparing of the sensory regions (like the primary visual and auditory cortex) [14]. It has been concluded that decreases in the whole-brain, entorhinal cortex, hippocampus and temporal lobe volumes are predictive of the progression of mild cognitive impairment to Alzheimer’s disease [13]. Given these findings and considering obesity a risk factor for cognitive impairment and dementia, this systematic review aims to take a deeper look into the present state of the art about the association between obesity and a reduction in GMV. It should be considered that comorbidities strongly linked to obesity [4], such as hypercholesterolemia, hypertension, type 2 diabetes and metabolic syndrome, have been found to be associated with an increased risk of dementia and lower brain volume [15,16,17,18,19,20]. Therefore, finding a significant association between GMV and obesity could be explained by a confounder factor’s effect on those comorbidities and not obesity itself. In addition, other variables not related to obesity, like age, sex and smoking status [16], could also act as confounding factors. Thus, to properly evaluate the role of obesity as an independent risk factor for brain volume reduction, the application of an accurate confounder’s adjustment should be necessary in every study in order to better understand the main results [21]. Nevertheless, several explanations are possible to clarify the link between obesity and GMV, and it is unclear whether previous volume brain abnormalities lead to overeating and obesity or if GMV reductions are a consequence of obesity [22]. In this sense, an increased accumulation of body fat (mainly in the abdominal cavity) can induce metabolic alterations such as insulin resistance, dyslipidemia, increases in oxidative stress or low-grade chronic inflammation [23]. In addition, the effects of aging in brain tissue could intensify by the accelerated expansion capacity of adipose tissue [24] and also higher body mass index (BMI) values have been related to increased prospective declines in gray matter volume over 5 years [25]. Neuropsychological and functional MRI studies have been performed and suggested that obesity was associated with lower inhibitory control exclusively over food-related responses [26], the hypofunction of inhibitory control regions and the increased response of food reward regions [27] and proposed a causal relationship between a lack of inhibition and overeating [28]. Following this idea while being against considering obesity a risk factor of reduced brain volume, Yokum et al., (2012) [29] and Pannacciulli et al., (2006) [22] released the first results concluding that it is gray matter reduction in the areas involved in taste (anterior insula, frontal operculum and Rolandic operculum), reward (orbitofrontal cortex, caudate and putamen) and inhibitory control (inferior, middle and superior frontal gyri), which could lead to abnormal eating behavior and promote obesity. Furthermore, as happens in drug-addicted subjects, obese individuals show reduced dopamine D2 receptors in the striatum, and the same neural pathways that reinforce natural appetitive behaviors are also activated in response to addictive drugs [30,31]. Regarding the brain differences found in obese or overweight people compared with individuals of normal weights, recent findings support the excess reward theory, which postulates that individuals at risk for obesity initially show hyper-reactivity of their reward circuits to high-calorie food tastes, which drives elevated intake of such foods. Furthermore, the results support the theory that an initial deficit in inhibitory control and a bias for immediate reward contribute to the excessive consumption of high-calorie foods. Consequently, these findings would imply that interventions should be aimed at reducing the responsiveness of reward regions and attention to food cues and increasing inhibitory control to reduce overeating and excessive weight gain [32].

A better understanding of the association between obesity and the GMV is necessary, as it may explain the role of obesity as a risk factor for mild cognitive impairment and dementia. To date, there are two recent meta-analyses which studied gray matter changes in obesity [33] and their relationship with executive function [34]. Moreover, a recent systematic review [35] addressed the association between executive dysfunction and obesity and being overweight over a lifespan. In our systematic review, we focused exclusively on the association between obesity and a pattern of changes in the global and local GMV, assessed by magnetic resonance imaging (MRI), including voxel-based morphometry (VBM) and cortical thickness (CT) and their association with cognition. The images of the reviewed studies were analyzed by VBM and CT, two fully automated unbiased methods which can examine the entire brain rather than a particular structure in an unbiased and objective manner and have the potential to detect changes that are difficult to detect by visual assessment or manual tracing [36]. Therefore, the main aim of this review is to evaluate the existence of a relation between obesity and GMV, assessed by MRI, and address the question of whether obesity is a relevant risk factor that could produce changes in the GMV. To this end, a systematic review of the existing literature has been carried out.

## 2. Materials and Methods

### 2.1. Research Strategies

A search was conducted in Pubmed, SCOPUS and Cochrane for those publications from inception until 1 February 2021 using the following keywords: (obes * OR adiposity OR leptin OR adiponectin OR (body mass index) OR (waist circumference) OR (waist-hip ratio)) AND ((voxel-based morphometric study) OR (CT) OR (brain volume) OR (cerebral atrophy) OR (gray matter)), and 1420 results were obtained. Additionally, this systematic review was performed according to the PRISMA statement [37]. There were two independent reviewers for screening and data extraction (M.F.-A. and E.M.), and any disagreements were resolved by a supervisor (N.G.-C.).

### 2.2. Eligibility Criteria

Only articles strictly related to humans written in Spanish or English were included in the assessment. The titles and abstracts of the publications were screened for relevance, and the following inclusion and exclusion criteria were considered. Only those publications which included original information were selected, except case report-type studies. Thus, duplicated studies, review articles, meta-analyses, intervention studies, editorials and letters were excluded. The studies could be cross-sectional or longitudinal and could include a control group. Those publications which included obese, overweight and lean participants without a diagnosis of mild cognitive impairment or dementia were considered. Studies which included in their sample mild cognitively impaired or demented patients (presenting pathologies like Alzheimer’s disease or vascular dementia) were excluded.

Only those studies including participants above or equal to 12 years of age were included.

The samples could be comprised of participants with comorbidities related or not related to obesity, such as cardiovascular diseases, type 2 diabetes, hypertension and hyperlipidemia, or healthy groups (without cardiovascular diseases or other comorbidities, as well as an absence of treatment for those conditions). Most studies included participants with comorbidities which were included as, for example, confounder’s adjustments in the majority of the articles (see Supplementary Table S1). However, 12 publications included a completely healthy sample in their assessment. Additionally, articles which were exclusively focused on participants with systemic or psychiatric diseases like schizophrenia, mania, major depression, anorexia nervosa, bulimia nervosa, polycystic ovary syndrome, AIDS or substance abuse were excluded.

### 2.3. Obesity Measurements

Regarding the types of evaluations, obesity measurement and confounder adjustment, obesity was measured by the body mass index, waist circumference, waist-to-hip ratio, plasma leptin levels, adiposity (measured as body fat percentage and fat mass index), visceral fat mass, ratio and volume and visceral adipose tissue. In addition, obesity had to be the essential variable assessed as a potential risk factor for change in GMV. Thus, studies which focused mainly on other comorbidities as principal covariates rather than obesity (i.e., those regarding solely patients with pathologies like diabetes mellitus, insulin resistance or metabolic syndrome) were excluded, as well as those which included patients with obesity-related or unrelated comorbidities and did not apply the confounder adjustment afterward.

### 2.4. Neuroimaging Technique and Analysis

The publications accepted were those which included MRI as the selected neuroimaging technique to study the GMV in relation with obesity. Exclusions were made for those studies which did not include neuroimaging or chose neuroimaging techniques different from MRI, assessed solely the white matter volume or evaluated the gray matter intensity or microstructural damage instead of the volume.

Regarding neuroimaging analysis, the GMV was evaluated with VBM or CT, excluding those publications which used other methods of volumetric segmentation. The technique of VBM maps gray matter loss on a voxel-by-voxel basis after an anatomic standardization. This is one of the objective and simple methods to avoid dependence on an a priori hypothesis and to adopt the principle of data-driven analysis [38]. Estimation of the CT, based on MRI T1-weighted images, provides a methodological option for volumetric assessments of cortical changes in the brain. The first image processing parts necessary to measure the CT involve segmentation of the images into gray matter, white matter and CSF, as occurs with VBM. Later, the posterior steps may take notably different processing methods in order to generate an absolute measure of the thickness across the cortical surface. Commonly, CT measurements include detection of the inner and outer cortical boundaries or surfaces, and this may be obtained using image and surface geometry information to get a representation of the brain’s gray and white matter surfaces [39,40].

Furthermore, studies selecting spectroscopy for gray matter assessment were excluded, since this method does not evaluate the volume but the cerebral metabolite concentration within the gray matter. Studies could include behavioral or neuropsychological assessments in their methods.

## 3. Results

### 3.1. Study Selection

**Figure 1:**
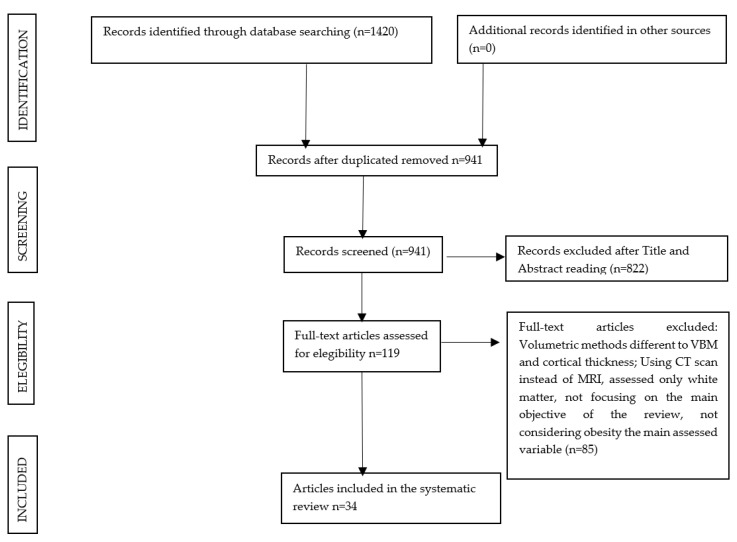
Flowchart of the search process for the systematic review.

The inclusion and exclusion criteria were applied to the titles and abstracts, and 941 publications were initially selected. In a second phase, a detailed assessment of the full-text publications was performed, and after applying the same eligibility criteria, 34 studies were finally included in this systematic review. The flow diagram of the study selection is represented in Figure 1.

### 3.2. Study Characteristics

Important information from each study was selected, and this is reported in Table 1, which is composed of the following items: (1) the year and first author of the study, ordered by the date of publication, (2) the study design, (3) the number of participants and main characteristic of the samples, (4) the mean age with the standard deviation and age range in years (data extracted from the whole sample, although in case the general data were not released but group-categorized, only the obese group data were used), (5) the type of obesity measurement (information for each variable was collected as the range or mean value with the standard deviation divided by subgroups), (6) the MRI magnet field strength (measured in tesla) and type of neuroimaging analysis, (7) the type of behavioral or cognitive assessment, in case cognition was evaluated (cognitive assessment was only reported for those studies which used this information in their results, excluding the data of articles applying evaluations solely to exclude demented participants from their study) and (8) results of the study. As shown in Table 1, the publications were released between July 2006 and February 2021, and the sample size in each study varied from 32 to 2344 participants. A total of 11 studies had samples exclusively comprised of healthy participants [7,22,41,42,43,44,45,46,47,48], and two studies included a healthy subgroup [49,50]. The main characteristics of the participants are shown under “Sample Size, Type of Groups” in the table.

In relation to the obesity measurement, 29 studies included the BMI as the type of obesity evaluation [6,7,8,9,25,42,43,44,45,46,50,56,59,60,61,62,63,64,66], and in 15 of them, the BMI was the only obesity-related measurement [6,7,8,9,21,22,25,29,41,42,45,52,54,55,56,57,59,60,62,64,66]. World Health Organization standards consider underweight, normal weight, pre-obesity, obesity class I, obesity class II and obesity class IIII BMIs to be below 18.5, 18.5–24.9, 25–29.9, 30.0–34.9 and 35.0–39.9 and above 40 kg/m^2^, respectively. However, three studies used the BMI for Asian populations instead of the regular BMI, considering underweight, normal weight, overweight, mild obesity and moderate-severe obesity BMIs to be below 18.5, 18.5–22.9, 23–24.9 and 25–27.4 and above 27.5 kg/m^2^, respectively [53,54,60]. The number of studies and type of obesity evaluation were as follows: 2 studies evaluated the waist-to-hip ratio [57,61], 4 studies used the waist circumference (cm) [44,46,60,61], 3 studies used the plasma leptin level (ng/mL) [48,63,65] (in 2 of them, it was the exclusive obesity measurement [48,65]), 2 studies used the body fat percentage (%) [47,49,57], 1 study used the fat mass index (kg/m^2^) [47] as the level of adiposity, one study used the visceral adipose tissue (I) [43] and 1 study evaluated the visceral fat mass (g) and volume (cm^3^) as the exclusive obesity measurement [58]. One study used the BMI, visceral fat ratio (VFR) (obtained by dividing the area of the intraabdominal fat by the total area of the abdominal cavity) and hepatorenal gradient (value obtained by subtracting the echogenicity of the hepatic region of interest (ROI) and the renal ROI) [51]. Comorbidities strongly linked to obesity and other variables not related to it, such as age, sex, smoking and drinking status or the total brain volume, could act as confounding factors and modify the association between the obesity measures and GMV. For this reason, all selected studies carried out the confounder adjustment. One of the review’s exclusion criteria was the diagnosis of mild cognitive impairment or dementia in the participants of the sample. However, it is interesting to assess the possible relation between obesity, GMV and cognition in case subtle cognitive impairment was related to the GMV loss in relation to obesity. Thus, 11 articles included behavioral or neuropsychological assessment in their methods [6,7,8,9,49,52,55,56,57,58,59]. As was said above, 34 studies were included in this systematic review, and regarding the chosen neuroimaging analysis, there were 21 VBM studies and 13 CT studies, whose results are about to be exposed.

### 3.3. VBM Studies

Regarding the studies which selected VBM as their neuroimaging analysis, 18 cross-sectional studies, 2 prospective longitudinal studies and 1 mixed study (including both cross-sectional and prospective longitudinal designs) were found.

#### 3.3.1. Cross-Sectional Design

In relation to the type of obesity measurement and the differences found after the gender-stratified analysis (in case it was applied), four studies demonstrated that a higher BMI was correlated with GMV reductions [6,9,41,46,50,53], and four studies found that obese participants had lower GMVs in comparison with the lean controls [22,44,55,59,62,63]. Those affected regions were the prefrontal, hippocampal and other structures belonging to the frontal and temporal cortex, namely the cerebellum, insula, parietal and occipital cortex, in addition to a less marked relation within the basal ganglia, thalamus, amygdala and limbic system. Lou et al. (2014) showed greater GMVs within the bilateral putamen compared with the control group [44]. However, two studies showed that a higher BMI was associated with a lower GMV within the bilateral putamen [49] and left putamen [54]. Taki et al. (2008) showed a negative correlation between the BMI and the GMV and the gray matter ratio (percentage of GMV in the intracranial volume) within all lobes, the anterior cerebellum and the midbrain and a positive correlation within regions of the frontal and temporal lobes, posterior cerebellum, thalami and caudate heads in men (no significant association was described in women) [57], and another study found that there was no significant association between the BMI and brain volumes (measured by total brain volume and GMV) [61].

Three publications concluded the existence of a relationship between a higher waist circumference and a lower GMV [46,50,53,61], and one study mentioned above showed a positive correlation between those variables within the bilateral putamen [57]. Debette et al. (2014) evaluated the correlation between the waist-to-hip ratio and the total brain volume and GMV and showed strong negative associations which were especially extensive in women. However, no association was found after confounder adjustment between the waist-to-hip ratio and hippocampal volume in women [61]. One study showed that higher plasma leptin levels were correlated with decreased GMVs within the insula [63], while Narita et al. (2009) described a positive association within the hippocampal cortex and cerebellum, and no significant association was described between the leptin level and total GMV [65]. Another publication showed higher GMVs within the cerebellum and inferior temporal gyrus and lower GMVs within the inferior frontal operculum, postcentral gyrus and putamen in relation to increased plasma leptin levels [48]. One healthy sample publication showed no significant association between adiposity (measured by fat mass and body fat percentage) and the GMV when adjusting for the free fat mass [47].

Regarding gender-stratified analysis, as well as what was said by Debette et al. (2014) [61], two more articles revealed significant negative correlations for the WC and GMV. The associations were similar for males and females; however, females showed more extensive negative correlations for the waist circumference and GMV [46,53].

In relation to neuropsychological assessment, one publication, despite demonstrating the negative association between the BMI and GMV, found negative associations between the BMI and memory performance and processing speed and found no significant association after the confounder adjustment when the evaluation of the executive function in relation to the BMI was made [6]. However, Walther et al. (2010), which also demonstrated the negative association between the BMI and GMV, found lower executive function in the obese group in comparison with the controls [9]. Shott et al. (2015) concluded that in the control group, the GMV of the gyrus rectus at the medial edge of the orbitofrontal cortex predicted a functional taste reward learning response in several regions but not in the obese group [59]. Additionally, in the work of Zhang et al. (2017), despite there being no significant difference between the obese and healthy subjects in the GMV of the OFC, the GMV of the OFC was negatively correlated with the hunger rating, relating to increased subjective motivation for eating behavior [54]. Wang et al. (2017), who studied impulsivity by means of the UPPS-P Impulsive Behavior Scale—including sensation seeking, perseverance, premeditation, negative urgency and positive urgency—showed that in the obese group, sensation seeking was negatively correlated with the GMV in the left amygdala and right pallidum [55].

#### 3.3.2. Prospective Longitudinal Design

Of the two longitudinal studies which analyzed the GMV via VBM, Brooks et al. (2013) evaluated a sample comprised of 75-year-old participants, which were followed up for 5 years and showed reductions in the global GMV and a lower GMV within the wide frontal and parietal cortex in the obese group in comparison with the controls. Furthermore, in relation to cognition, the obese group showed lower executive function [8]. Yokum et al. (2012) performed a study with a female adolescent sample which was followed for a year and concluded that the BMI and GMV were negatively correlated within the frontal cortex [29].

#### 3.3.3. Prospective Longitudinal Cross-Sectional Design

Bobb et al. (2014) developed a study with both prospective longitudinal and cross-sectional designs with an older adult sample. The cross-sectional regions of interest and VBM demonstrated that a higher BMI was related to a lower GMV in the whole cortex and within the frontal lobes, respectively. Meanwhile, prospective longitudinal regions of interest showed a negative association within the temporal and occipital cortex [25].

### 3.4. CT Studies

Regarding the studies which selected the CT for their neuroimaging analysis, 12 cross-sectional studies were found, and 1 article had both cross-sectional and longitudinal designs.

#### Cross-Sectional Design

In relation with the type of obesity measurement and the differences found after the gender-stratified analysis (in case it was applied), three studies showed lower GMVs in the obese group in comparison with the lean controls [7,45,64], and two studies concluded the existence of a negative correlation between the CT and BMI [42,43,49], especially within the orbitofrontal cortex and other regions in the prefrontal and frontal cortex, in addition to the insula, temporal, parietal and occipital cortex. One of those studies also demonstrated that higher visceral adipose tissue was related with reduced CT [43]. Ronan et al. (2016) concluded that there was no relation between the BMI and the regional CT and cortical area surface. However, an increased mean CT was found in the overweight and obese subjects in comparison with the lean controls [56].

Saute et al. (2018) [51] found that measures of the CT did not differ between obese and lean adolescents, nor was it associated with the BMI or hepatorenal gradient. This study revealed that adolescents with higher rates of intra-abdominal fat had greater cortical thickness in several brain regions (including the fusiform gyrus, insular cortex and precentral and postcentral gyri). In addition, De Groot et al. (2017) [52] showed that adolescents with obesity had larger pallidum volumes compared with the lean adolescent group. In the obese group, a larger pallidum volume was positively associated with the ability to delay reward in the Choice Delay Task. Another study evaluating the relationship between the BMI in the Asian population and CT demonstrated increased CT in the overweight and mildly obese male groups compared with the controls, but no significant association was found in the moderate–severe obesity group. The BMI and CT showed no significant association either [60]. One study also showed that a higher visceral fat mass and volume was correlated with increased CT [58]. Kim et al. (2015) demonstrated the existence of a positive correlation between the body fat percentage and CT and a negative correlation between the waist-to-hip ratio and CT in men, but no significant association was found in women [57].

In relation to neuropsychological assessment, four studies carried out the evaluation of cognition, and three of them showed no significant association between obesity and cognitive performance [56,57,58]. However, Yau et al. (2014) showed that the obese group had lower academic achievement (arithmetic and spelling), working memory, attention, psychomotor efficiency and mental flexibility [7]. On the other hand, Westwater et al. [49] found that in both adolescents and adults, a greater BMI and temporal discounting of monetary reward (i.e., a greater valuation of smaller, immediate rewards over larger, delayed rewards, evaluated using the Monetary Choice Questionnaire) was related to lower left triangular IFG thickness. This author suggests that greater impulsive tendencies in higher BMI individuals may be explained in part by structural alterations in the prefrontal cortex. Finally, De Groot et al. (2017) [52] found that a greater pallidum volume was positively associated with the inhibitory control, specifically the number of large rewards in the Choice Delay Task in the group with obesity but not in the lean participants. In addition, they found a corresponding association; however, it only trended toward significance between the pallidum volume and the stop signal reaction time in the stop signal task (adjusted for age and sex), indicating that better inhibitory control is associated with greater volume of the pallidum only in the participants with obesity. The authors speculate that greater pallidum volumes in obesity might be a neural adaptation to loss over inhibitory control and that greater pallidum volumes in the group with obesity indicate a more successful adaptation, leading to more adequate executive function.

## 4. Discussion

Obesity shows high rates of worldwide prevalence, and it has become a major public and individual problem due to its link with multiple systemic conditions, such as greater cognitive impairment and dementia [10,11,12]. This systematic review has analyzed and summarized the existing literature related to the association between obesity and GMV, assessed by magnetic resonance imaging (MRI) and analyzed by VBM or CT.

Most studies found a significant association between obesity and lower global and regional GMVs, especially within the prefrontal, hippocampal cortex and other regions located in the frontal and temporal lobes. In addition, a lower GMV was also found in the parietal and occipital cortex, cerebellum, insula, basal ganglia, thalamus, amygdala, limbic lobes and other regions [6,7,8,9,25,29,42,43,44,45,46,48,50,57,59,61,62,63,64,66]. Many of these areas have been shown to have decreased connectivity specifically in the parietal cortex, posterior cingulate and prefrontal cortex after a program with a Mediterranean diet and physical activity in obese people [67]. In addition, lower connectivity in the posterior cingulate and lateral inferior parietal posterior cingulate cortex, involved in the coordination of self-referential thinking and internal state subsystems such as appetite or other gut signal processing or food-related cognitive factors, was also reported in obese people [68]. Furthermore, in the study by Garcia-Casares et al. (2017) [67], a decrease was also seen, among other areas, in the connectivity between the prefrontal cortex and the insular gyrus, connections that would reflect the ability to assess the perception of hunger during fasting, an aspect altered in obesity. In addition, the insular cortex seems to be related to cognitive and emotional control, termination of intake and the ability to predict the satisfactory effects of food intake [69].

These findings strengthen the role of obesity as a risk factor for cognitive impairment and dementia, as the hippocampal and temporal cortex are the main structures affected in the early stages of Alzheimer’s disease, and other mentioned regions are also altered during the disease’s progression [13]. The prefrontal cortex is related to the executive function, inhibition of inappropriate responses and regulation of taste, reward and behavioral processing, playing a part in the regulation of eating behavior, so a volume reduction in the frontal and prefrontal cortex could interfere with an obese person’s ability to predict future consequences of their eating behaviors, making them prone to aberrant overeating and promoting obesity [22,45,61]. In addition, the main limitations of previous studies were assessed to develop a better and more accurate design, choosing both VBM and region of interest as the neuroimaging analysis methods. The comparison of the images after the follow-up suggested that a higher body mass index may lead to a progressive reduction in GMV within the temporal and occipital cortex, including the hippocampus. The cross-sectional assessment also found a strong association between the body mass index and a lower GMV within the bilateral frontal cortex. The temporal and hippocampal abnormalities may be redolent of the pattern of gray matter changes seen in Alzheimer’s disease [25]. However, although most of the assessed studies reported a negative association between the body mass index and global and local GMVs, the altered regions were variable and widespread between studies. Moreover, some articles showed contradictory results, such as no significant [47,51,60,61] or positive associations between obesity and the GMV within certain brain regions [44,48,51,52,54,56,57,58,60,65,66]. Taki et al. (2008) concluded the existence of a positive association between the body mass index and bilateral putamen, and due to its significant role in the food reward system, it may motivate abnormal dietary intake and the development of obesity [57]. This result matches the aforementioned GMV reduction in the prefrontal cortex found in several studies, as both alterations could lead to overeating and obesity. On the other hand, the nucleus accumbens and other mesolimbic areas also seem to play a key role in the pathophysiology of obesity, although the results are heterogeneous, probably due to confounding variables such as the amount of visceral body fat, MRI analysis software or scanner types. In a recent meta-analysis [70], it was found that, in general, the volume of the nucleus accumbens was positively related with the BMI. However, the latter depended on the variable of age, such that for younger people, this relationship would be positive, whereas for older adults, it would be negative. The authors concluded that the increase in the volume of the nucleus accumbens at a young age could be a risk factor for obesity in adulthood, in which the decrease in the volume of the nucleus accumbens would be related to a higher BMI. Consequently, this fact could be due to the prolonged effect of neuroinflammation in the brain [70].

Kim et al. (2015) found that in men, there was a negative correlation between the waist-to-hip ratio and CT, but a positive correlation was found between the latter variable and body fat percentage. The possible explanation could be that association between obesity and the GMV might be caused by body fat distribution rather than the fat amount, and an increase in the body fat percentage could be related to the presence of neuroprotector factor leptin and adiponectin, leading to a greater GMV instead of lowering it [57]. The results of Narita et al. (2009) agreed with the latter ones when finding that a higher plasma leptin level was related to an increased GMV within the hippocampus and cerebellum, hypothesizing the neuroprotective effects of leptin on human brain aging [65]. However, the existence of opposite results in relation to the leptin levels [48,63] points out the need for further studies evaluating the implications of leptin levels in GMV abnormalities. The importance given to the confounder adjustment in the introduction of the review was proven in the results, as some studies found different patterns of gray matter distribution after applying the gender-stratified analysis. Furthermore, Taki et al. (2008) also reported that visceral fat predominates in men while subcutaneous fat predominates in women, and this dissimilar fat distribution could be related to different GMV alterations between the sexes [57].

Eleven articles also evaluated a behavior or cognition [6,7,8,9,49,52,55,56,57,58,59], and supporting previous studies, most VBM studies concluded a lower executive function in obese people in comparison with lean adults, as well as lower memory performance, processing speed, academic achievement, working memory, attention, psychomotor efficiency and mental flexibility [8,9,59]. However, many associations evaluated in the CT studies remained non-significant [56,57,58]. When this systematic review was carried out, the following limitations were found in the design: the sample size and type of evaluations of the assessed studies. On the one hand, the selected articles were heterogeneous in their designs, as it was found that most of them (*n* = 30 studies) were cross-sectional, while only four publications had a prospective longitudinal design (two of them had both cross-sectional and prospective longitudinal designs, being the only study which performed two obesity and neuroimaging assessments, happening before and after the follow-up [25]). The latter design could be more appropriate, as it can study the temporal changes in the global and regional GMVs in obese subjects, which would help examine the cause–effect relationship and determining if obesity is an independent risk factor for GMV reduction (as is suggested in most assessed publications [7,9,21,25,41,43,46,48,49,53,55,57,58,61,62,65]) or if, on the contrary, previous volume brain abnormalities lead to overeating and obesity, results initially suggested by Yokum et al. (2012) [29] and Pannacciulli et al. (2006) [22] and mentioned in the introduction and later by posterior studies [8,42,44,50,59,63]. Masouleh et al. (2016) even proposed that obesity and gray matter changes might have caused each other reciprocally [9]. In relation to cross-sectional design, although most results concluded a significant negative association between obesity and the GMV, it is not possible to determine the timeline of the development of the observed differences.

Therefore, further prospective longitudinal studies which perform multiple image acquisitions are needed to elucidate the real sequence of events, because a better understanding of the association between obesity and the GMV may explain the role of obesity as a risk factor for cognitive impairment and dementia. In relation to the design, there were also huge differences in the sample sizes of the studies, varying from 32 to 2344 participants, and there were 18 publications including less than 100 participants in their samples. Regarding the obesity measurement, most studies (*n* = 29) included the body mass index as an obesity variable. However, the body mass index does not reflect fat distribution within the body, and it has been suggested that abdominal fat distribution appears to be more closely correlated with adverse effects and brain volume alterations than global body mass measures like the body mass index [46,53,57,61]. For this reason, studies selecting more accurate adiposity measurements like the waist circumference or waist-to-hip ratio are necessary, as prevention programs aimed at reducing abdominal fat could prevent potential GMV abnormalities and dementia until older ages. The studies’ heterogeneity can also be noticed in the neuroimaging analysis performed, finding 21 studies using VBM and only 13 using CT. These techniques provided different gray matter measurements, and studies using both to analyze a single dataset have reported differences in the results, attributed to biology and methodology [71,72]. Additionally, VBM is a nonbiased, whole-brain automated technique that detects the regional volume differences of white and gray matter using structural images without prior hypotheses regarding specific ROIs [37]. On the other hand, ROI analysis was then applied to the smoothed brain images, and gray and white matter values were extracted for each participant in a particular brain ROI. The values describe the proportion of gray or white matter within the selected voxel and thus represent an estimate of the gray or white matter volume [73]. The results of VBM investigations have reported evidence of a much more complete pattern of volumetric differences in the brains of obese people that include both larger and smaller cortical matter volumes. These analysis differences between the selected studies for this review could represent a limitation for obtaining conclusions that should be considered for future research on this topic. The MRI scanners and magnet field strength also varied, and although most publications used MRI machines with 1.5 or 3 tesla (8 and 23 studies, respectively), and one longitudinal study used on the first assessment a 1.5-tesla MRI and a 3-tesla scanner after the 5-year follow-up [25], two studies had magnet field strengths of 0.5 and 1 tesla [21,64].

Most studies did not include a neuropsychological assessment in their evaluation, while the role of obesity as a risk factor for both cognitive impairment and GMV reduction could be better assessed with studies including both neuropsychological and structural techniques. Those heterogenous factors could have caused the high variability in the results of the studies, finding widespread changes in specific regions of the GMV between studies and positive or non-significant associations between obesity and the GMV.

On the other hand, the main strength of this article is that a very systematic evaluation has been performed. Keywords were carefully selected, strict inclusion and exclusion criteria were applied in two phases (to the title and abstract on the one hand and to the full-text publications in the second phase), and analysis and data extraction of each of the 34 studies were summarized into the main table of this review (Table 1).

## 5. Conclusions

Obesity is associated with lower global and regional GMVs, especially within the frontal and temporal lobes, although other variables and widespread regions have been found. Further prospective longitudinal studies considering the distribution of adiposity, carrying out correct confounder’s adjustment and including an extensive neuropsychological assessment are needed to achieve a better understanding of the association between obesity and the GMV, as it may explain the role of obesity as a risk factor for cognitive impairment and dementia. Thus, the high worldwide prevalence and its significant consequences for individual and public health highlight the importance of developing public health programs and interventions, because the prevention of obesity would imply not only reduced obesity-related comorbidities and risk of cardiovascular disease, but also decreased risk of developing GMV reduction, cognitive impairment and dementia until older ages.

## Figures and Tables

**Table 1:** Summarized data of the studies included in the review. Articles are shown in chronological order.

Year and First Author	Study Design	Sample Size, Type of Groups	Total Sample Mean Age (Unless Indicated): M ± SD (Range)	Obesity Measurement	MRI Analysis Description	Neuropsychological Assessment	Results
2019, Franz CE [21]	Prospective longitudinal	*n* = 373 Obese (O) vs. Control (C) (171 vs. 202)	O= 61.7 ± 2.65 C= 62.0 ± 2.48 The reported data are given by the 2 groups separately	BMI (4 time measures according to normal/O trajectories) 32.6 ± 3.00 vs. 25.9 ± 2.48 The reported data are given by the 2 groups separately	1T MRI CT ROI analysis	No	Obesity trajectory had thinner cortex compared with the normal trajectory in the superior, inferior, middle temporal gyri, temporal pole, fusiform gyrus, superior temporal sulcus, frontal pole, pars triangularis, caudal and rostral middle frontal gyri.
2019, Westwater ML [49]	Cross-sectional	Adolescents = 70 O vs. Overweight vs. Healthy weight adolescents (18 vs. 17 vs. 35) Adults = 75 O vs. Overweight vs. Healthy weight adults (20 vs. 21 vs. 34)	Adolescents = Women (W)_:_ 35, 16.7 ± 1.4; Men (M): 35, 16.4 ± 1.5 Adults _=_ 42W, 32.8 ± 6.3; 33M, 33.9 ± 6.4 The reported data are given by groups and genders separately (W/M).	BMI: Adolescents: 32.3 ± 2.4 vs. 26.7 ± 1.4 vs. 21.2 ± 2.1 Adults: 33.5 ± 2.6 vs. 27.6 ± 1.6 vs. 22.1 ±1.7 Body Fat (%) Adolescents: 31.5 ± 9.4 vs. 23.4 ± 10.2 vs. 16.1 ± 7.8 Adults: 34.6 ± 8.8 vs. 29.3 ±7.7 vs. 20.4 ± 6.1 The reported data are given by groups and genders separately (W/M)	3T MRI CT ROI analysis	Impulsivity (delay discounting (DD)): monetary choice questionnaire	In adolescents, increasing age-adjusted BMI Z-score attenuated age-related CT reductions globally and in frontal, temporal and occipital regions. In adults, an increase in BMI increased age-related CT reductions both globally and in bilateral parietal cortex. Increased DD and adiposity were associated with a reduction in IFG thickness in adolescents and adults.
2018, Saute RL [51]	Cross-sectional	*n* = 44 O vs. Lean (L) (18 vs. 26)	O: 16.22 ± 0.73 L: 16.81 ± 0.71 The reported data are given by the 2 groups separately	BMI: 31.11 ± 3.21 vs. 21.38 ± 1.70 VFR: 14.62 ± 7.15 vs. 5.10 ± 1.70 HRG: 14.44 ± 8.44 vs. 10.48 ± 7.72 The reported data are given by the 2 groups separately	3T MRI CT whole-brain analysis	No	No relationship between BMI or HRG with brain cortical dimensions. Significant positive correlation between VFR and cortical areas in left paracentral, lateral occipital and right precentral, postcentral and superior temporal, among others. CT reductions were not different between groups, neither were they associated with BMI or HRG.
2017, de Groot CJ [52]	Cross-sectional	*n* = 44 O vs. L (23 vs. 19)	Total sample age range: (12–16)	BMI: Significantly higher BMI in the obesity group (−0.08 vs. 3.54 standard deviation values)	3T MRICT ROI analysis	Executive function: stop signal task and Choice Delay Task	O group had greater significant volumes of the pallidum when compared with C. A greater pallidum volume in O group was positively significant, associated with delay reward in the Choice Delay Task.
2017, Opel N [41]	Cross-sectional	Healthy groups from two different studies: *n* = 330 (Münster Neuroimaging Cohort) and *n* = 347 (BiDirect study)	Münster Cohort = 39.2 ± 11.3 (20–59) BiDirectstudy = 51.6 ± 8.2 (35.3–65.6) The reported data are given by the 2 groups separately	BMI: 24.5 ± 3.9 vs. 26.3 ± 4.1 The reported data are given by the 2 groups separately	3T MRI VBM whole-brain analysis	No	Higher BMI and PR for obesity (single-nucleotide polymorphisms (SNPs) selected from the genome-wide association study (GWAS)) were significantly associated with decreased medial prefrontal gray matter, and it was further demonstrated that prefrontal gray matter significantly mediated the effect of PR for O on the BMI in both samples.
2017, Hayakawa YK [53]	Cross-sectional	*n* = 792 healthy sample (M = 523, W = 269)	M: 55.3 ± 9.7 (23–84) W: 55.2 ± 9.9 (24–81) The reported data are given by gender groups separately (W/M)	BMI 24.7 ± 3.1 vs. 22 ± 3.3 WC 88.5 ± 8.1 vs. 81.2 ± 9.8 The reported data is given by gender groups separately (W/M).	3T MRI VBM whole-brain analysis	No	Negatively significant correlations were found between WC and BMI and gray matter volume. In W, the total area of the regions (left thalamus, precentral and inferior frontal gyrus) was significantly correlated with WC and was slightly larger than that of the regions significantly correlated with BMI.
2017, Zhang B [54]^4^	Cross-sectional	*n* = 40 O vs. L (20 vs. 20) Men adult sample	O: (20–28) L: (20–28) The reported data are given by the 2 groups separately	BMI: 33.56 ± 3.53 vs. 21.48 ± 1.43 The reported data is given by the 2 groups separately.	3T MRI VBM whole-brain analysis	Hunger rating: visual analog scales	O group in comparison to C: increased GMV in the left putamen and it was positively correlated with BMI, plasma insulin and HOMA-IR. There was a negative correlation between OFC GMV and hunger score and no differences between groups.
2017, Wang H [55]	Cross-sectional	*n* = 80 O vs. C (31 vs. 49)	O: 39.58 ± 1.93 vs. C: 29.55 ± 1.41 The reported data are given by the 2 groups separately	BMI: 34.38 ± 0.69 vs. 21.87 ± 0.29 The reported data is given by the 2 groups separately.	3T MRI VBM ROI analysis	Impulsivity: UPPS-P Impulsive Behavior Scale, eating behavior: Three-Factor Eating Questionnaire and depression (Beck Depression Inventory-II)	O group in comparison to C: reduced GMV in the frontal and limbic regions. In the O group: sensation seeking was negatively correlated with GMV in the left amygdala and right pallidum.
2016, Ronan L [56]	Cross-sectional	*n* = 473 O/Overweight vs. Normal weight (77/150 vs. 246)	54 (20–87)	BMI: O: 33.5 ± 3.8 vs. Overweight: 27.1 ± 1.6 vs. Normal weight: 22.7 ± 1.7	3T MRI CT whole-brain analysis	Cognitive performance: Cattell Culture Fair	No relation between BMI with cortical area surface, regional CT and cognitive performance. Overweight and O groups in comparison to C: increased mean CT.
2016, Medic N [42]^4^	Cross-sectional	*n* = 202 O vs. Overweight vs. C Healthy sample 19 smokers	32.3 ± 7.7 (18–50)	BMI: 28.5 ± 6.3 (18.5–46.4)	3T MRI CT whole-brain analysis	No	BMI with global CT: not significant association. BMI with regional CT: negatively correlated within left LOC and right vmPFC.
2016, Masouleh SK [6]	Cross-sectional	*n* = 617 No C group	68.7 ± 4.6 (60–79)	BMI: 27.5 ± 4 (18.5–45.5)	3T MRI VBM whole-brain analysis	Alzheimer diagnosis (CERAD plus): TMTA and B, semantic and phonemic verbal fluency and verbal memory	BMI with GMV: negatively correlated within the prefrontal, temporal, insular and occipital cortex, thalamus, putamen, amygdala and cerebellum. BMI memory performance: negatively associated within the prefrontal cortex, thalamus, orbitofrontal cortex and insula and paracingulate gyrus due to its effect in the GMV. BMIs with processing speed were negatively correlated after the adjustment for almost all confounders within the bilateral temporal and insula and intracalcarine cortex.
2015, Kim HJ [57]	Cross-sectional	*n* = 1777 O vs. C (M = 312, W = 174) Gender-stratified analysis Reported data are for the groups and gender, separately (F/M)	M: 64.9 ± 7.0 (45–91) W: 62.6 ± 7.5 (45–85) Reported data are for the groups and genders separately (F/M)	Body fat % and WHR: M: 24.0 ± 5.4 and 0.937 ± 0.036, W: 32.0 ± 6.0 and 0.913 ± 0.057 Reported data is for the groups and gender, separately (F/M)	3T MRI CT whole-brain analysis	Cognitive performance and dementia diagnosis: MMSE and MoCA	M: body fat % with CT: positively correlated in all brain lobes; WHR and CT: negatively correlated in total and in the frontal lobe. Low fat % and central obesity related to decreased CT in men but not to MMSE or MoCA. W: no relation between CT and body fat % or WHR. No relation between body fat % and WHR and the MMSE or MoCA.
2015, Janowitz D [50]	Cross-sectional	Study of Health in Pomerania (SHIP-2) = 758 and SHIP-TREND = 1586 Healthy group from SHIP-2 and SHIP-TREND (*n* = 435)	SHIP-2: 49.8 ± 9.3 SHIP-TREND: 46.3 ± 11.3(20–79) The reported data are given by the 2 groups separately	WC and BMI: SHIP-2: 88.7 ± 12.8 and 27.4 ± 4.5 SHIP-TREND: 87.9 ± 12.6 and 27.2 ± 4.4 The reported data is given by the 2 groups separately	1.5T MRI VBM whole-brain analysis	No	WC with GMV: negatively correlated, especially within the frontal lobe and thalamus. BMI and gray matter: negatively associated. In the healthy subsample, WC with GMV: negatively associated within right gyrus rectus, bilateral frontal lobes, left insula, temporal and cerebellum.
2015, Kaur S [58]	Cross-sectional	*n* = 103 No C group	49.63 ± 6.47 (40–60)	VFM and VFV: 1276.33 ± 1021.17 1206.93 ± 961.73	3T MRI CT whole-brain analysis	Global cognition (MMSE, Vocabulary and Matrix WASI II subtests and full-scale IQ), Memory (CVLT-II), executive function (TMTA and B, COWA, WAIS III Digit Span and Stroop color–word subtest)	VFM and VFV with CT: positively correlated within the right posterior cingulate gyrus. VFM and VFV with cognitive assessment: no significant association.
2015, Shott ME [59]	Cross-sectional	*n* = 42 O vs. C (18 vs. 24) W sample (neuroimaging carried out during the first 10 days of the follicular menstrual phase)	O: 28.7.4 ± 8.3 C: 27.4 ± 6.3 The reported data are given by the 2 groups separately	BMI: 34.78 ± 4.44 vs. 21.64 ± 1.26	3T MRI VBM whole-brain analysis	Neuropsychiatric assessment: depression (Beck Depression Inventory (BDI)]; novelty seeking, reward dependence and harm avoidance, (Temperament and Character Inventory (TCI)); drive for thinness, body dissatisfaction, bulimia, (Eating Disorder Inventory-3 (EDI-3)); punishment sensitivity, reward sensitivity (Revised Sensitivity to Punishment and Reward Questionnaire (SPSRQ)) and state and trait anxiety (Spielberger State and Trait Anxiety Inventory (STAI))	O group in comparison to C: lower GMV within the OFC, striatum and insula. In C group, GMV of the gyrus rectus at the medial edge of the OFC predicted functional taste reward-learning response in frontal cortex, insula, basal ganglia, amygdala, hypothalamus and anterior cingulate cortex, but not in the O group.
2015, Kim H [60]	Cross-sectional	*n* = 1111 O mild/moderate–severe vs. Overweight vs. C vs. Underweight (260/114 vs. 313 vs. 406 vs. 18) Gender-stratified analysis Reported data are for the groups and gender, separately (F/M)	M: 64.7 ± 7.0 W: 62.1 ± 7.3 (≥45)	BMI for Asian population: moderate to severe obesity ≥27.5 vs. 25–27.4 mild obesity vs. 23–24.9 overweight vs. 18.5–22.9 normal weight vs. <18.5 underweight	3T MRI CT whole-brain analysis	No	M, underweight: lower CT in the frontal and temporal regions; overweight: greater CT in the frontal cortex; mildly O: greater CT in the frontal, temporal and occipital. No significant difference in moderate–severe obesity. W: BMI and CT: no significant association.
2014, Veit R [43]	Cross-sectional	*n* = 72 O/Overweight vs. Normal weight (13/17 vs. 42) Healthy sample	29.65 ± 8.15 (19–50)	BMI and VAT: 25.49 ± 5.18 (17.70–46.49) and 2.39 ± 1.56 (0.31–7.58)	3T MRI CT whole-brain analysis	No	BMI and VAT with CT: negatively correlated within left occipital area, inferior temporal cortex and precentral and inferior parietal areas. VAT with CT: negatively correlated in the right insula, the left fusiform gyrus and the right inferior temporal.
2014, Yau PL [7]	Cross-sectional	*n* = 53 O vs. C (26 vs. 27) Healthy sample (adolescents)	O: 17.64 ± 1.62 (14.89–20.76) C: 17.22 ± 1.55 (14.28–20.21) The reported data are given by the 2 groups separately	BMI: 35.47 ± 5.88 vs. 21.12 ± 2.18	1.5T MRI CT whole-brain analysis	Intellectual functioning and academic achievement (Vocabulary and Matrix Reasoning WASI subtests and WRAT), memory (WRAML), executive function (TMTB, Tower of London Test (TOL), Wisconsin Card Sorting Test (WCST), Stroop Test and COWAT), attention and psychomotor speed (WRAML, TMTA, Digit Vigilance Test (DVT) and Digit Symbol Substitution Test (DSST))	O group in comparison with C: lower academic achievement (arithmetic and spelling), working memory, attention, psychomotor efficiency and mental flexibility, CT within the OFC and ACC.
2014, Debette S [61]	Cross-sectional	*n* = 1779 Obesity tertiles (T1: control)Gender-stratified analysis	72.8 ± 4.1 (≥65)	WHR, BMI and WC: 0.87 ± 0.09 and 25.4 ± 3.8 and 86.4 ± 12.2	1.5T MRI VBM whole-brain analysis	No	M and W, WHR with TBV and GMV: negatively correlated; WC and GMV: negatively correlated. More extensive correlations in W. BMI with brain volumes (TVB and GMV): no significant association. Women, WHR with HV: no association after confounder’s adjustment.
2014, Bobb JF [25]	Cross-sectional Prospective longitudinal (5 years)	*n* = 347 O/Overweight vs. C (142/158 vs. 47) L workers + healthy = Single group	O: 59.8 ± 7.3–64.7 ± 7.4	BMI: O: 33.1 ± 2.9 vs. Overweight: 27.7 ± 1.4 vs. C: 23.5 ± 1.2 (21.0–43.3)	1.5T–3T MRI VBM whole-brain and ROI analysis	No	Cross-sectional, BMI with GMV: negatively correlated in ROI and VBM. Longitudinal, BMI with GMV: negatively correlated within the temporal and occipital cortex in ROI.
2014, Lou B [44]	Cross-sectional	*n* = 49 O/Overweight vs. C (22 vs. 27) Healthy sample (Chinese young adults)	O/Overweight: 31.72 ± 8.04C: 29.04 ± 7.32 The reported data are given by the 2 groups separately	BMI and WC: 31.44 ± 3.34 vs. 21.54 ± 2.06 and 100.25 ± 9.97 vs. 76.91 ± 7.70	3T MRI VBM whole-brain analysis	No	O/Overweight group in comparison with C group: lower GMV within the left prefrontal cortex, bilateral cingulate gyrus and the right temporal lobe and greater GMV within the bilateral putamen. BMI and WC with GMV: positively associated within bilateral putamen.
2013, Marqués-Iturria I [45]	Cross-sectional	*n* = 37 O vs. C (19 vs. 18) Healthy sample	O: 33.7 ± 5.7 C: 32.3 ± 5.9 (20–40) The reported data are given by the 2 groups separately	BMI: 36.08 ± 5.92 vs. 22.54 ± 1.94 (30.1–49.69 vs. 19.53–24.97)	3T MRI CT whole-brain analysis	No	O group in comparison with controls: reduced CT within left superior frontal and right medial OFC; ventral diencephalon and brainstem volumes.
2013, Kurth F [46]	Cross-sectional	*n* = 115 O (*n* = 11) vs. Overweight (*n* = 11) vs. Normal vs. Underweight Gender-stratified analysisHealthy Caucasian sample	45.17 ± 15.45 (18–80)	BMI and WC: 25.02 ± 4.13 (18.18–42.37) and 0.87 ± 0.12 (0.67–1.32)	1.5T MRI VBM whole-brain analysis	No	BMI and WC with GMV: negatively correlated within hypothalamus, prefrontal, anterior temporal and inferior parietal cortex and cerebellum (more widespread and pronounced for WC). W: more extensive correlations for WC than M.
2013, Brooks SJ [8]	Prospective longitudinal (5 years)	*n* = 292 O vs. C (59 vs. 97)	75 (70–75)	BMI: 33.0 ± 0.3 vs. 22.5 ± 0.2	1.5T MRI VBM whole brain-analysis	Executive function: Trail Making Test	O group in comparison with C: lower global GMV and GMV within bilateral supplementary motor area, bilateral DLPFC, left inferior frontal gyrus and postcentral gyrus and lower executive function (related with DLPFC).
2013, Weise CM [47]	Cross-sectional	*n* = 76 O vs. Overweight vs. C (36 vs. 8 vs. 36) Healthy sample	32.1 ± 8.8	FFMI and adiposity (Body fat % and FMI): 8.4 ± 5.6 and (25.5 ± 10.9 and 21.4 ± 3.8)	1.5T MRI VBM whole-brain analysis	No	FFMI with GMV: negatively correlated within bilateral temporal regions, the bilateral medial and caudolateral OFC and the left insula independent of adiposity. Adiposity with GMV: no significant association when adjusted for FFM.
2012, Mueller K [62]	Cross-sectional	*n* = 43 O/Overweight vs. C (27 vs. 16) NSE levels (neuronal injury marker) Young adults	O/Overweight: 26.4 ± 5.4 (20–41)	BMI O/Overweight: 33.0 ± 6.4 (25.3–50.7)	3T MRI VBM whole-brain analysis	No	O/overweight group in comparison with C: lower GMV and elevated serum NSE levels within the hippocampal cortex and the cerebellum.
2012, Smucny J [63]	Cross-sectional	*n* = 53 O-prone (overweight) vs. O resistant (lean C) (28 vs. 25)	O-prone (overweight): 30.29 ± 3.81 O resistant: 31.32 ± 3.45 (25–40)	BMI and plasma leptin level: O-prone (overweight): 26.2 ± 2.0 vs. O resistant: 21 ± 2 and O-prone (overweight): 11.42 ± 7.27 vs. O resistant: 3.91 ± 2.89	3T MRI VBM whole-brain and ROI analysis	No	O-prone group in comparison with C: lower GMV within the insula, medial OFC and cerebellum. Leptin level and measures of hunger with GMV: negatively correlated within the insula.
2012, Yokum S [29]	Prospective longitudinal (1 year)	*n* = 83 O vs. Overweight vs. Normal (17 vs. 31 vs. 36) W-adolescent sample	O: 18.4 ± 2.8	BMI: 17.3–38.9	3T MRI VBM whole-brain and ROI analysis	No	1-year-change BMI with GMV: negatively correlated within bilateral superior frontal and left middle frontal cortex.
2012, Hassenstab JJ [64]	Cross-sectional	*n* = 53 O vs. Successful weight loss maintainers vs. Never-O lean (C) (17 vs. 17 vs. 19)	O: 47.8 ± 7.6 (27–65)	BMI: 34.0 ± 3.6 vs. 23.7 ± 1.5 vs. 21.7 ± 1.9	3T MRI CT ROI analysis	No	O group in comparison with the never-O lean group: lower CT within DACC, PPC and AIC. Successful weight loss maintainers: intermediate results between both O and never-O lean groups, with lower CT within the PPC compared to the never-O lean group.
2010, Walther K [9]	Cross-sectional	*n* = 95 O vs. Overweight vs. Normal weight (20 vs. 22 vs. 53) Older-W sample	O: 66.9 ± 9.9 (52–92)	BMI: 34.9 ± 3.3 vs. 27.6 ± 1.4 vs. 22.3 ± 1.6	3T MRI VBM whole-brain and ROI analysis	MMSE, vocabulary test from WASI, memory (WMS-III, WMS-R and CVLT); executive function (WCST; WAIS-R; verbal fluency task, WMS-III and processing speed (TMTA))	BMI with GMV: negatively correlated within the left orbitofrontal, right inferior frontal, precentral gyri, parahippocampal, fusiform and lingual gyri and cerebellar regions. O group: lower executive function.
2009, Narita K [65]	Cross-sectional	*n* = 34 Asian population BMI > 27 excluded Gender-stratified analysis	M: 64.8 ± 5.1 (57–76) W: 64.3 ± 4.4 (56–74)	Plasma leptin level (ng/mL): M: 3.1 ± 1.7 W: 4.9 ± 1.9	3T MRI VBM whole-brain analysis	No	Leptin level with total GMV: no significant association. Leptin level with GMV: positively correlated within the right hippocampus and bilateral cerebellum (leptin acts as a neuroprotective hormone in those regions). M and W separately: no significant associations.
2008, Taki Y [66]	Cross-sectional	*n* = 1428 O (*n* = 27) vs. Overweight (*n* = 273) vs. Normal vs. Underweight Gender-stratified analysis Japanese sample Reported data is for the groups and gender, separately (F/M)	M: 44.5 ± 16.1 W: 46.4 ± 14.1 (12–81)	BMI: M_:_ 23.41 ± 3.00 (13.2–40.1) W: 22.23 ± 2.97 (15.2–33.3)	0.5T MRI VBM whole-brain analysis	No	M, BMI with GMR (GMV/ICV): negatively correlated; with GMV: negatively correlated within all lobes, anterior cerebellum and midbrain and positively correlated within frontal and temporal lobes, cerebellum, thalami and caudate. W, BMI with GMR and GMV: no significant association.
2007, Pannacciulli N [48]	Cross-sectional	*n* = 32 O vs. C (16 vs. 16) Caucasian participants Healthy sample	32 ± 9 (18–49)	Plasma leptin level (ng/mL): 16 ± 20 (0.5–65)	1.5T MRI VBM whole-brain analysis	No	Plasma leptin levels and GMV: positively correlated within the left cerebellum and inferior temporal gyrus and negatively correlated within the left inferior frontal operculum, postcentral gyrus and right putamen.
2006, Pannacciulli N [22]	Cross-sectional	*n* = 60 O vs. C (24 vs. 36) Non-diabetic Caucasian participants Healthy sample	O: 32 ± 8 L:33 ± 9	BMI: 39.4 ± 4.7 vs. 22.7 ± 2.2	1.5T MRI VBM whole-brain analysis	No	O group in comparison with C: lower GM density within the post-gyrus, frontal operculum, putamen and middle frontal gyrus.

ACC: anterior cingulate cortex; AIC: anterior insular cortex; BDI: Beck Depression Inventory; Body fat %: body fat percentage; BMI: body mass index (kg/m^2^); C: control; CERAD plus: Consortium to Establish a Registry for Alzheimer’s disease; CI: confidence interval; COWAT: Controlled Oral Word Association Test; CT: cortical thickness; CVLT-II: California Verbal Learning Test; DACC: dorsal anterior cingulate cortex; DD: delay discounting; DLPFC: dorsolateral prefrontal cortex; DSST: Digit Symbol Substitution Test; DVT: Digit Vigilance Test; EDI-3: Eating Disorder Inventory-3; FFM: fat-free mass; FFMI: fat-free mass index (kg/m^2^); FMI: fat mass index (kg/m^2^); GMR: gray matter ratio; GMV: gray matter volume; HOMA-IR: homeostasis model assessment of insulin resistance; HRG: hepatorenal gradient; HV: hippocampal volume; IFG: inferior frontal gyrus; L: lean; LOC: left lateral occipital cortex; M: men; MCC: midcingulate cortex; MMSE: Mini-Mental State Examination; MoCA: Montreal Cognitive Assessment; NSE: neuron-specific enolase; O: obese; OFC: orbitofrontal cortex; PPC: posterior parietal cortex; PR: polygenic risk; ROIs: regions of interest; SPSRQ: Revised Sensitivity to Punishment and Reward Questionnaire; STAI: Spielberger State and Trait Anxiety Inventory; T: tertil; TCI: Temperament and Character Inventory; TOL: Tower of London Test; TMT: Trail-Making Test; UPPS-P Impulsive Behavior Scale: Urgency, Lack of Premeditation, Perseverance, Sensation Seeking, Positive Urgency, Impulsive Behavior Scale; VAT: visceral adipose tissue; VBM: voxel-based morphometry; VFM: visceral fat mass (g); VFR: visceral fat ratio (%); VFV: visceral fat volume (cm^3^); vmPFC: ventromedial prefrontal cortex; W: women; WAIS III: Wechsler Adult Intelligence Scale; WAIS-R: Wechsler Adult Intelligence Scale Revised; WASI: Wechsler Abbreviated Scale of Intelligence; WC: waist circumference (cm); WHR: waist-to-hip ratio; WMS-III: Wechsler Memory Scale; WMS-R: Wechsler Memory Scale Revised; WRAML: Wide Range Assessment of Memory and Learning; WRAT: Wide Range Achievement Test; WCST: Wisconsin Card Sorting Test.

## Data Availability

No new data were created or analyzed in this study. Data sharing is not applicable to this article.

## Supplementary Materials

The following are available online at https://www.mdpi.com/article/10.3390/brainsci11080999/s1, Table S1: Summarized confounder’s adjustment of the studies included in this review. Articles are shown in chronological order.
