# Supplementary material for: Obesity and Gray Matter Volume Assessed by Neuroimaging: A Systematic Review
Source: Brain Sci. 2021 Jul 28;11(8):999. doi: 10.3390/brainsci11080999 (PMC8391982; doi:10.3390/brainsci11080999)
Supplement: Supplementary file 1 [file brainsci-11-00999-s001.zip › brainsci-1271053-supple.pdf]

Table S1. Summarized confounder's adjustment of the studies included in this review. Articles are shown in chronological order.

YEAR, FIRST AUTHOR	CONFOUNDER'S ADJUSTMENT
2019, Franz CE [21]	Age, lifetime education, ethnicity, smoking status and being at-risk for hypertension, dyslipidemia, DM, inflammation, and/or ischemic heart disease at Time 4.
2019, Westwater ML [49]	Age, sex, education, age-adjusted BMI Z-score.
2018, Saute RL [51]	Age, sex and TIV.
2017, de Groot CJ [52]	Age, sex and false discovery rate.
2017, Opel N [41]	Age, sex
2017, Hayakawa, YK [53]	Age, MMSE, TIV, BMI and WC.
2017, Zhang B [54]	-
2017, Wang H [55]	Age, sex and handedness
2016, Ronan L [56]	Age, sex, educational level, income; current smoking (not included as a controlled parameter due to low number of smokers, with similar distribution between groups), physical activity; SPB, DBP; myocardial infarction, cancer, DM, stroke, hypercholesterolemia, HBP, cognitive impairment; TIV, gray-white-matter contrast.
2016, Medic N [42]	Age, sex.
2016, Masouleh SK [6]	Age, sex; Mean SPB, smoking status, depression, educational level; HBP or antihypertensive medication, DM (type 1 or 2), antihyperlipidemic medication, estrogen supplements, cardiovascular diseases; white matter hyperintensities; APOE e4-alleles.
2015, Kim HJ [57]	Age, educational level, smoking status, alcohol consumption; SPB, DBP, fasting blood sugar, cholesterol level, postmenopausal state; HBP, DM, hyperlipidemia, previous stroke, cardiovascular disease, intracranial volume
2015, Janowitz D [50]	Age, sex; SBP, DBP, HBP or antihypertensive medication, DM type 2 or antidiabetic medication, LDL, HDL, total cholesterol.
2015, Kaur S [58]	Age, SBP, total cholesterol, blood glucose level
2015, Shott ME [59]	Age
2015, Kim H [60]	Age, education level, hypertension, DM, hyperlipidemia, previous stroke, cardiovascular disease, smoking status, drinking and smoking status, SBO, DBP, fasting blood sugar, cholesterol, ICV.
2014, Veit R [43]	Sex, age, educational level, total surface area; BMI (for VAT).
2014, Yau PL [7]	Sex, ICV, hypertension.
2014, Debette [61]S	Age, gender, ICV, SPB, antihypertensive treatment, smoking, DM, LDL, HDL, log-transformed triglycerides, lipid lowering treatment, history of vascular disease
2014, Bobb JF [25]	Race, ApoE4 status, age, smoking status, ICV, BMI
2014, Lou B [44]	Age, sex.

2013, Marqués-Iturria I [45]	Age, educational level
2013, Kurth F [46]	Age, sex.
2013, Brooks SJ [8]	Sex, BMV, educational status, DM.
2013, Weise CM [47]	Age, sex and handedness.
2012, Mueller K [62]	Age, total GMV, serum soluble leptin receptor level.
2012, Smucny J [63]	Age, sex, and ICV.
2012, Yokum S [29]	Initial BMI, 1-year-change BMI, total GMV.
2012, Hassenstab JJ [64]	Age, sex, antihypertensive medication
2010, Walther K [9]	Age, ICV, hypertension.
2009, Narita K [65]	Age, sex, BMI, WHR, ICV.
2008, Taki Y [66]	Age, sex, drinking status, hypertension, DM
2007, Pannacciulli [48]N	Age, sex, % body fat, fasting plasma insulin concentrations, global GMV.
2006, Pannacciulli N [22]	Age, sex, handedness, global tissue density.

APOE: Apolipoprotein E; Body fat %: Body fat percentage; BMI: Body mass index (kg/m2); BMV: Brain matter volume; DBP: Diastolic blood pressure; DM: Diabetes mellitus; FFMI: Faf-free mass index (kgm2); FMI: Faf mass index (kgm2); GMV: Gray matter volume; HBP: High blood pressure; ICV: Intracranial volume; M: Men; MMSE: Mini-mental State Examination; MoCA: Montreal Cognitive Assessment; NSE: Neuron-specific enolase; P: Percentile; P16: percentile 16; Q: Quartil; Qi: Quintil; SAT: Subcutaneous adipose tissue; SD: Standard deviation; SPB: Systolic blood pressure; T: Tertil; TIV: Total intracranial volume. VAT: Visceral adipose tissue; VFM: Visceral fat mass (g); VFV: Visceral fat volume (cm3); W: Women; WC: Waist circumference (cm); WHR: Waist-to-hip ratio.
